# Biomechanical Study of the Osteoporotic Spine Fracture: Optical Approach

**DOI:** 10.3390/jpm11090907

**Published:** 2021-09-11

**Authors:** Mircea Sopon, Valentin Oleksik, Mihai Roman, Nicolae Cofaru, Mihaela Oleksik, Cosmin Mohor, Adrian Boicean, Radu Fleaca

**Affiliations:** 1 Orthopaedic-Traumatology Surgery Department, Sibiu Emergency Clinical Hospital, 550024 Sibiu, Romania; mirceasopon@yahoo.com; 2 Department of Industrial Machinery and Equipments, Lucian Blaga University of Sibiu, 550024 Sibiu, Romania; 3 Department of Surgery, Lucian Blaga University of Sibiu, 550024 Sibiu, Romania; mihai.roman@ulbsibiu.ro (M.R.); radu.fleaca@ulbsibiu.ro (R.F.); 4 Department of Industrial Engineering and Management, Lucian Blaga University of Sibiu, 550024 Sibiu, Romania; nicolae.cofaru@ulbsibiu.ro (N.C.); mihaela.oleksik@ulbsibiu.ro (M.O.); 5 Department of Basic Science, Lucian Blaga University of Sibiu, 550024 Sibiu, Romania; cosmin.mohor@ulbsibiu.ro; 6 Department of Medicine, Lucian Blaga University of Sibiu, 550024 Sibiu, Romania; adrian.boicean@ulbsibiu.ro

**Keywords:** biomechanics, osteoporosis, disc degeneration, strain, wedge fracture, optical approach

## Abstract

Background and objectives: Osteoporotic spine fractures represent a significant factor for decreasing quality of life in the elderly female population. Understanding the mechanisms involved in producing these fractures can improve their prevention and treatment. This study presents a biomechanical method to produce a vertebral fracture, conducted on a human spine segment, observing the displacements and strains in the intervertebral disc, endplate, and vertebral body. Materials and Methods: We performed two tests, one corresponding to an extension loading, and the second to an axial loading. Results: The maximum displacement in the target vertebral body presented higher values in the case of the extension as compared to the axial strain where it mainly occurred after the fracture was produced. The strains occurred simultaneously on both discs. In the case of the axial strain, due to the occurrence of the fracture, the maximum value was recorded in the spine body, while in the case of the extensions, it occurred in the neural part of the upper disc. The advantage of this method was that the entire study was an experiment, using optical methods, increasing the precision of the material data input. Conclusions: The research method allowed recording in real time of a larger amount of data from the different components of the spine segment. If there was an extension component of the compression force at the moment of the initial loading, part of this load was absorbed by the posterior column with higher mechanical resistance. After the maximum capacity of the absorption was reached, in both situations the behavior was similar.

## 1. Introduction

Fractures represent one of the affections with most dramatic sequelae for elderly people. Spine fractures, in the case of elderly people, represent one of the main signs of osteoporosis, especially if they are accompanied by deformations of the spine, and chronic pains, resulting in decreasing the patient’s quality of life. These fractures are important for clinical activity, not only considering the significant decrease in the patient’s quality of life, but also the impact on health services. Fracture pattern and behavior are important considering location, type, and bone density. Once a patient has a fracture, the risk of having a second fracture is increased in the case of osteoporosis [1]. The fracture occurs when the mechanical resistance of the vertebra is inferior to the load on the segment to which it is subjected. A very important aspect in clinical activity is the fact that, although mineral bone density decreases evenly, there is a more frequent occurrence of spine fractures at the T7–T8 and T12–L2 levels [2]. This is mainly due to the biomechanics of the spine [3], vertebral micro-architecture changes [4], the transition from a stiff area to a mobile one, and native spine curves which generate increased loads in certain areas.

Flexion is one of the components that can be involved in producing spine fractures. In native-spine, live conditions, many fractures are produced by axial load on the spine, but there is a component of the force determined by the increase in the natural spine curves. During axial load, usually, there is a plastic deformation of the vertebrae body (which has a lower mechanical strength on axial loading compared with the posterior column), generating the deformation in angular flexion of the spine, localized at the siege of the fracture. The flexion component of the force becomes more important with deformation due to the fracture. In a lumbar spine, there is lordosis, which means that, on an axial load, the behavior of the spine may be to hyperextend, not to flex. However, in our experiment, we used a short spine segment and we studied the moment the fracture lines appeared on the vertebral body. Therefore, for our experiment, the flexion component of the load was not significant.

Considering lumbar lordosis, as mentioned before, on an axial load the spine segment can be compressed axially. The goal of our experiment was to investigate this and see whether, if the force applied was more posterior, the segment would hyperextend. In the last situation, the mechanical behavior change, and even if the initial behavior may be relevant (increasing native curves of the spine), it is not relevant for the compression fracture of the vertebral body (as is usual in osteoporotic fractures). This is the reproduced mechanism of a fracture produced by hyperextension.

The purpose of this study was to analyze the biomechanical behavior of the osteoporotic spine on pure axial loading and combined axial–extension loading and understand the mechanisms generating fractures of the vertebral body. A study on the occurrence of spine fractures is very important, but at the same time very difficult to conduct due to spine anatomy as well as the major differences between in vivo and in vitro requirements. This is the reason why views differ from one study to another, from using the finite-element method to the biomechanical method, on spine segments harvested from humans or pigs. For the current study, we adopted the biomechanical method on a human spine segment, observing the displacements and strains in the intervertebral disc, endplate, and vertebral body.

The importance of this study is related to understanding the behavior of the human spine in loading and the mechanism that generates an osteoporotic fracture. Understanding fracture mechanisms can provide important data that can be used to prevent these fractures, such as the load needed to produce the fracture, the way the deformations occur, and where is the siege of the initial deformation. All these data can be used to develop preventive measures and to improve therapeutic techniques, such as vertebroplasty. The fracture pattern generated by the axial load, even if it is in experimental conditions that do not entirely reproduce in vivo conditions, can therefore increase understanding of the fracture mechanism. As there is no possibility to study the development of a fracture pattern generated in vivo, experimental techniques are useful methods to achieve these goals.

## 2. Materials and Methods

**Figure 1:**
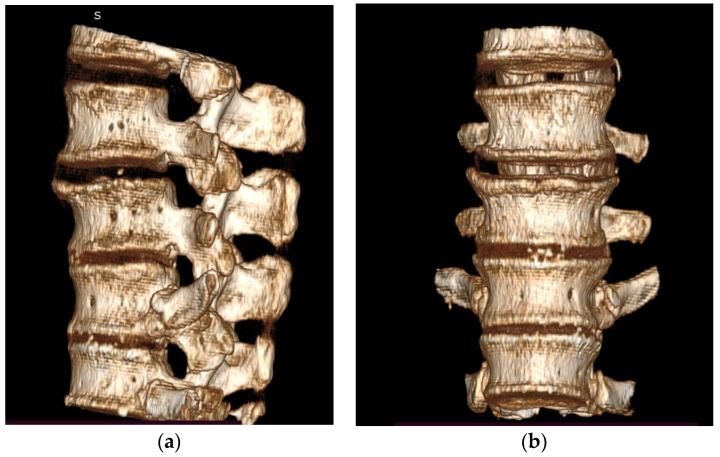
(**a**) CT image 3D reconstruction of the analyzed spine segment–lateral view; (**b**) anteroposterior view.

For the study, we harvested a human T12–L3 spine segment (age 75, female), and we studied the appearance of the fracture at the L1 vertebra. The preparation consisted of removing the muscular tissue, preserving the capsuloligamentous system of the spine segment (longitudinal anterior and posterior longitudinal ligaments, supraspinous and interspinous ligaments, intervertebral discs, articular processes). The harvested segment was preserved in a freezer at −20 °C temperature. The absence of any previous trauma was objectified by radiographies and Computer Tomography scanning after prelevation of the spine and before the biomechanical experiment (Figure 1). To evaluate bone mineral density, the Bone Mineral Density (BMD g/cm^2^) was estimated with osteodensitometry. The results were: T12 0.575 g/cm^2^ (T-score −4.1; Z score −3.0), L1 0.698 g/cm^2^ (T-score −4.2; Z score −3.2), L2 0.678 g/cm^2^ (T-score −3.8; Z score −2.6), corresponding to osteoporosis.

The experiment was conducted on an assembly of machines and devices consisting of: an Instron 5587 tensile-compression testing machine, an Aramis 2M optical measuring system for strains, and an experimental layout necessary to fix the spine segment on the machine. The testing machine was an Instron 5587 machine for mechanical testing. It was equipped with Bluehill 2 software used for both machine control and data processing. Considering the fact that the Instron 5587 tensile-compression testing equipment only allows the determination of the load–displacement curves for various tests, the Aramis 2M optical measuring system, equipment which was produced by GOM [5], was used to determine the main strains and the displacement of the spine segment components. This system offered the possibility of real-time measurement of the strains occurring in the compressed vertebral body. The optical measuring system was equipped with a software used to control the system, as well as to extract and process data. The main advantages of using such a system were the following: it offered 3D complete information regarding coordinates, displacements, strain distribution, etc.; it used a non-contact measuring method; the analysis was material-independent; high precision and local resolution (up to ±0.01%); it could be used for large displacements and strains (<100%); the number of images/sec could be adjusted: 1–20 Hz, 480 Hz, 960 Hz.

The spine segment subject in this study was covered with a thin layer of matt argent paint, to prevent undesired reflections. The spine segment was set for the paint to dry for a few minutes, then it was covered in black graphite powder. During compression testing, the black graphite points changed their coordinates. The acquisition system determined the coordinates of each point for every image acquired, through the optical measuring system, and then determined the displacements and the specific strains. The system could identify three types of specific strains: technical, logarithmic, and Green. The technical strains were chosen to be presented in this study as they were easier to compare to the results of the simulations performed using the finite-element method found in the literature. The experimental layout used for the compression tests of the spine segment was especially designed for this study, for the purpose of following the anatomic shape of the spine body, and at the same time allowing it to be fixed. The rack was designed to allow its mounting on the “T” channel base plate of the test machine.

The spine segment was loaded increasingly from 0 N to 2000 N, at room temperature, because a series of biomechanical studies were conducted at values between 2 and 14 kN [6,7,8]. The resulting data are represented by the load–displacement pair points collected by means of the data acquisition system of the test machine. For higher data accuracy, the pairs of load–displacement points were acquired at a rate of 200 pairs/second. The purpose of the study was to determine the behavior of the spine segment before, during, and after the occurrence of the fracture.

## 3. Results

**Figure 2:**
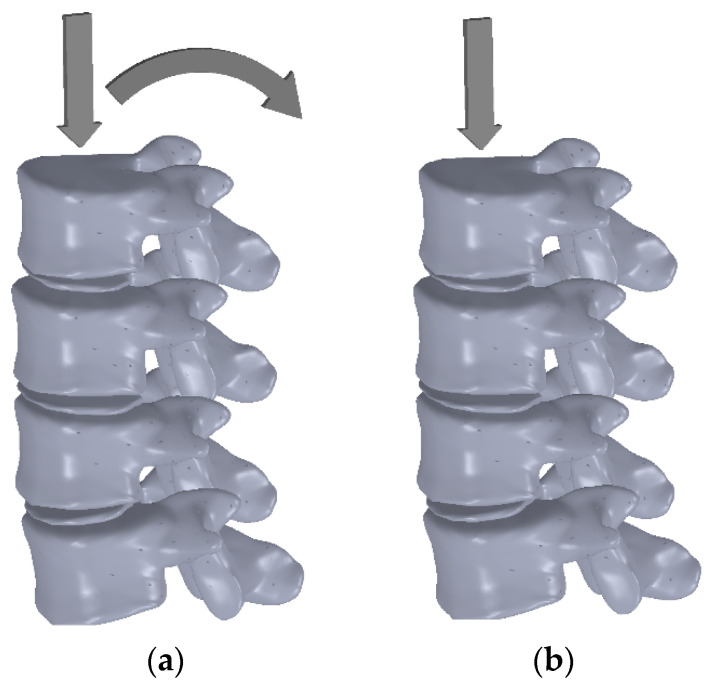
(**a**) Scheme for axial and extension load; (**b**) scheme for pure axial load.

Biomechanical tests with an increasing loading from 0 N to 2000 N were conducted to observe the frontal and side displacements of the spine segment. Two load tests were conducted, the first test consisting of the loading generated mainly on the neural elements at a 2 cm distance behind the rotation center, involving an extension component (Figure 2a), and the second test consisting of a pure axial loading, with pressure applied on a point corresponding to the rotation center (Figure 2b) [9].

**Figure 3:**
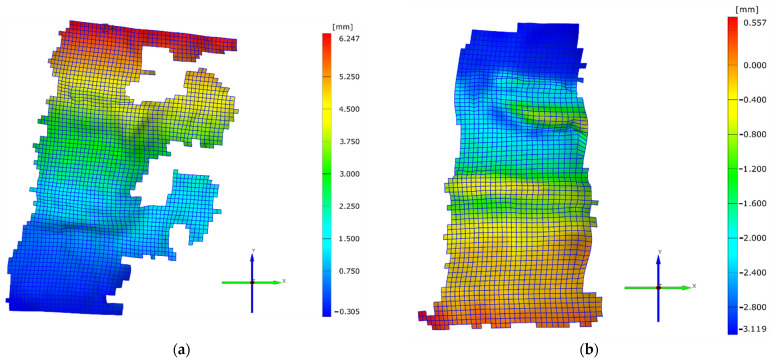
(**a**) Displacement values for the axial and extension case; (**b**) displacement values for the pure axial case.

This can be easily observed in Figure 3a,b, which present the displacement that occurred in the two compression tests at the end of the test, when the maximum 2000 N value was reached. The maximum backwards displacement of the vertebral body measured 6.25 mm in the case of the axial and extension, and 3.12 mm in the case of pure axial loading. However, in the case of the pure axial loading, the biggest displacement was observed immediately after the fracture was produced, due to the compaction of the L1 vertebral body.

The specific strains that were registered at the level of the intervertebral discs and the vertebral bodies were mainly observed. The target segments were the L1 vertebra as well as its upper and lower intervertebral discs.

**Figure 4:**
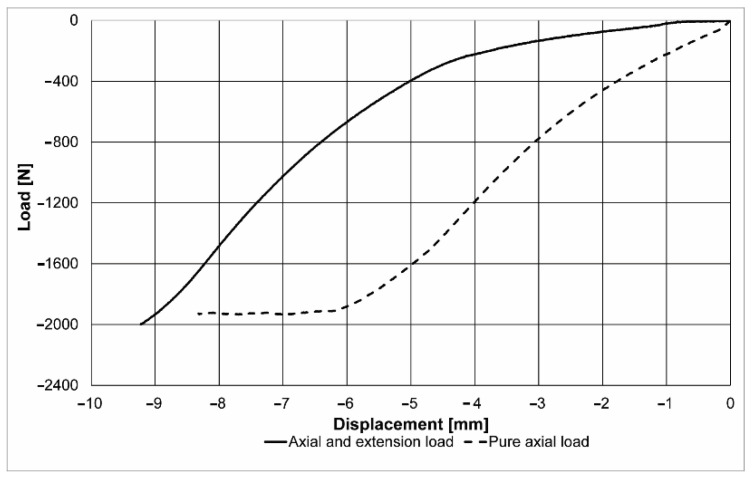
Load–displacement curves for the axial and extension and pure axial cases.

The graph in Figure 4 presents the load curves (displacement force) for the two loading tests. As can be observed, the maximum load was 2000 N in both cases, but the manner in which this value was achieved was different. The compression of the spine segment had a bending direction in the axial and extension loading (zone 1), while in the second case, it had an axial loading (zone 2). This is the result of the fact that the loading was generated in the neural area, and when these elements rotated (extension), the second stage could be observed, where the loading was distributed on the entire surface of the vertebral body. These results are demonstrated by the change of the slope of the load–displacement curve, of approximately 500 N, and by the moment when the loading type changed.

The second curve presents the graph of a pure axial loading, and the force was seen to increase faster as compared to the first case, because there was no possibility for the neural elements to rotate, this movement being locked by the articular processes. Once the wedge fracture occurred at a value about 1930 N, it could be seen that the load increased slowly, simultaneously with the displacement, probably due to specific strains in the case of osteoporosis at the level of the vertebral trabecular system. In the case of pure axial loading, the maximum displacement was nevertheless reduced compared to that in the case of the eccentric loading (extension), due to the increased stiffness of the spine segment in this position.

**Figure 5:**
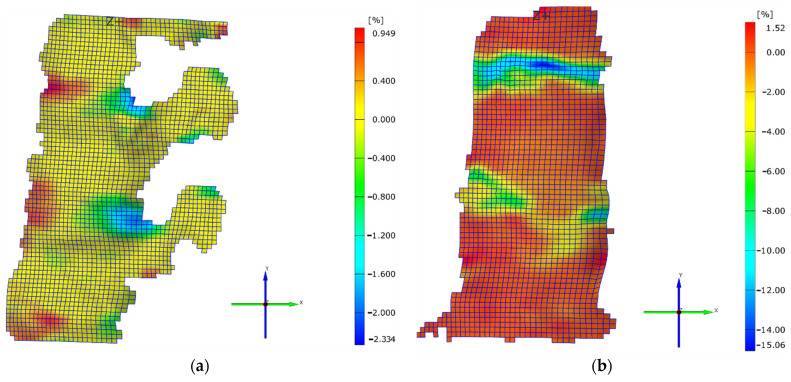
(**a**) Strain on Oy direction for the axial and extension case for a displacement value of 2.5 mm; (**b**) strain on Oy direction for the pure axial case for a displacement value of 2.5 mm.

During progressive loading, an increase in the strain value εy (axial strain) could be observed in both discs, in both axial and extension loading and pure axial loading. The maximum values in the case of the axial loading were obtained as shown in Figure 5a,b. Figure 5 presents the strain variation charts ε y for the two loading types at the same displacement value of the vertebral body (at 2.5 mm). The maximum strain values ε y registered values of −2.33% in the case of the axial and extension loading, and −15.1% in the case of the pure axial loading. The negative values were due to compression, and the difference between the two values was generated by the fact that, while in the case of the extension the vertebral body rotated and moved from the front to the back, in the case of the axial loading it could not change position and the stress was absorbed by the intervertebral disc. The graph in Figure 4 reveals that the load increased suddenly in this situation. In both cases, the suprajacent intervertebral disc registered a more important strain than the subjacent disc. Upon observation of the sample in axial and extension loading, an initial load of both discs, as well as of the neural elements, was noticed. Another fact that could be observed was that, during the first stage, due to the specific nature of the strain, in the case of the extension component, the anterior part of the intervertebral discs was not as stressed as the posterior part, but it was rather subjected to elongation, the strain value being a positive one (0.95%). This was determined by the displacement of the entire vertebral body, which rotated from front towards back during extension, the lower part of the segment being fixed. Thus, it can be explained why the inferior intervertebral discs, as well as the neural elements, were compressed in axial loading, while the posterior part was stressed in elongation.

In the case of both types of strain, it could be observed that the posterior part of the intervertebral disc was mainly loaded (Figure 5a,b). This aspect was also highlighted in other biomechanical studies conducted on cadavers, where a higher load of neural elements could be noticed, as compared to the anterior ones in case of elderly people who presented degenerative changes of the intervertebral disc [10,11].

**Figure 6:**
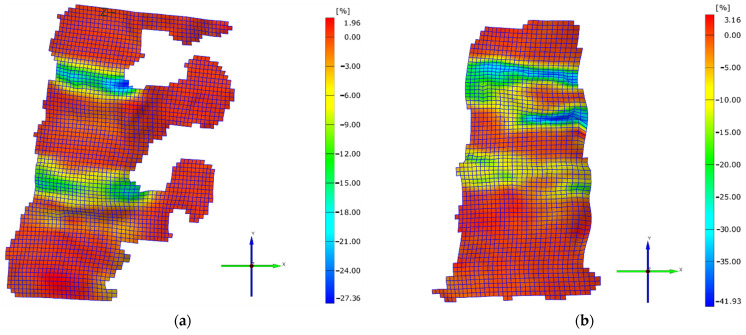
(**a**) Strain on Oy direction for the axial and extension case on the final loading value (2000 N); (**b**) strain on Oy direction for the pure axial case on the final loading value (2000 N).

When increasing the load value up to 2000 N, it could be observed that all the aspects mentioned above were confirmed, the higher strain values were registered by the suprajacent intervertebral discs in both loading cases, and the higher strain values were registered in the case of axial loading, as shown in Figure 6a,b. The maximum strain value εy was 27.36% in the case of axial and extension, and 41.93% in the case of the pure axial loading. In the case of the pure axial loading, due to the occurrence of the fracture, the maximum value was registered inside the vertebral body, while in the case of the axial and extension load, it was registered at the posterior part of the suprajacent intervertebral disc.

**Figure 7:**
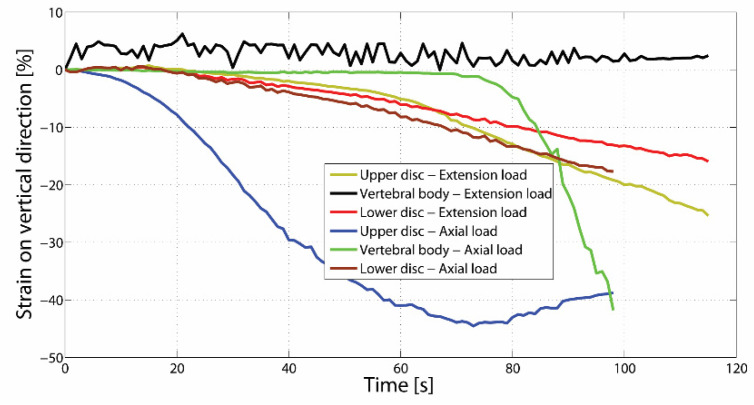
Strain variation between axial and extension loading and pure axial loading for six-points situated in the suprajacent intervertebral disc, subjacent disc, and vertebral body.

To compare the strain values in the superior intervertebral discs, the inferior discs, and vertebral body between the two types of loading, three points were selected for each type of compression (axial and extension case and pure axial), located in the maximum strain area on the Oy direction of the suprajacent disc, subjacent disc, and vertebral body. Figure 7 presents the variation graph for the strain value of the sixth-points during the tests. Thus, it could be easily noticed that for both the suprajacent disc and subjacent disc the maximum strain values were registered in the case of pure axial loading, and the strain values of the suprajacent disc were higher than those of the subjacent disc during tests in both types of compression.

During the axial and extension compression test, the same aspects were noticed as in the case of pure axial loading, but unlike these, the load of the neural elements was also noticed up to a certain value. When this value was reached, the progressive loading of the vertebral body and the intervertebral discs started. This was because, until a certain value, the load was supported and absorbed by the components of the neural arch and the articular processes.

During the axial and extension progressive loading, the increasing loading of the intervertebral discs could be noticed, the difference consisting of the fact that the suprajacent disc loaded at a higher value compared to the subjacent disc (Figure 7).

In order to identify the mechanism which generated the fracture, one point for each strain type in the maximum strain area on L1 vertebra was selected, apart from the two points mentioned above.

In the case of axial and extension loading, the maximum strain values εy continuously increased, having the same variation curve up to a certain value (17%), and from that point on the increase accelerated in the suprajacent intervertebral disc. It could be observed that the strain deformation in the vertebral body was close to zero.

**Figure 8:**
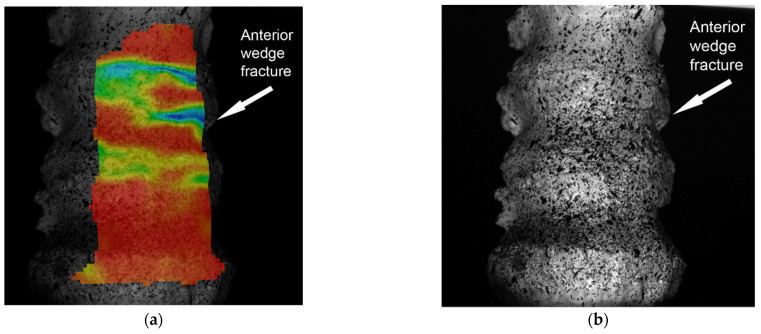
(**a**) The moment of wedge fracture-strain variation; (**b**) the moment of wedge fracture-acquired image without strain.

When analyzing the graph, it could be observed that, up to a certain peak value, an increasing loading of the intervertebral discs was registered (the suprajacent disc reached a strain value of approximately 30%, and the subjacent disc registered a value of approximately 20%). Afterwards, in the case of pure axial loading, an increase in the strain value εy was noticed, generating an increasing strain of the vertebral body. Thus, it resulted that, until a certain value, the load was absorbed by the intervertebral discs, but after their maximum strain degree was reached the load began to gradually be supported by the vertebral body. This began to load increasingly until reaching a peak value, and from that moment on, a failure of the trabecular system and the anterior wall of the vertebral body could be noticed. After reaching this value, the trabecular system of the vertebra started to cease, resulting in an anterior wedge vertebral body fracture, characterized by the compaction of the vertebral body and strains at the level of the vertebral plate (Figure 8a,b).

After the fracture appeared, a decrease in the intervertebral disc strain values taking over the load and distributing it towards the trabecular system of the vertebral body could be observed.

**Figure 9:**
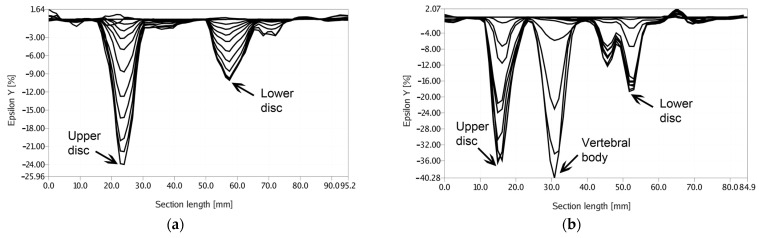
(**a**) Cross-sectional strain variations for the axial and extension load; (**b**) cross-sectional strain variations for the pure axial load.

To emphasize the behavior of the vertebral body within its assembly, a cross-sectional analysis was also realized, including the maximum strain areas for both pure axial loading and axial and extension load, and the behavior in time of this section of the vertebral body. The results of this analysis are presented in Figure 9a,b. Only 10 of the corresponding sections were selected, in order not to have a very crowded graph.

The results of this analysis show that in the case of the axial and extension load, there were two local maximum values corresponding to the intervertebral discs, while in pure axial compression, besides the two maximum values corresponding to the discs, the graph presents an overall maximum corresponding to the strain value of the vertebral body. Moreover, the maximum strain value in the suprajacent disc was observed to be approximately equal to the maximum strain value of the vertebral body.

## 4. Discussion

Structural integrity of the vertebral spine is highly important for quality of life and normal functions of the entire body. Unfortunately, degenerative changes caused by aging trigger severe changes to spine anatomy with irreversible changes to its response to activities of daily living. Intervertebral disc degenerative changes are generally present after the age of 50. The relation between the structure of the vertebral body and its strength is very complex, involving vertebral plates, the cortical system, and the trabecular system, as well as the intervertebral disc [12].

The intervertebral disc plays a very important part in taking over and absorbing different load forces and distributing them at the level of the vertebral plates. Almost every human action involves the spine. Therefore, it is extremely important to understand the biomechanical behavior of this structure. At the level of the vertebra, compressive forces are transferred from the level of the intervertebral disc to the level of the vertebral plate, and afterwards to the structure of the vertebral body (trabecular system and vertebral cortical). As a result of aging, there are biochemical changes at the level of the intervertebral disc and vertebral plate [13,14]. These changes have an important impact on how the vertebral segment is loaded in the case of elderly compared to young adults. Thus, according to biomechanical studies and the finite-element method, the stress is higher in the central area of the vertebral body [15,16] and in the posterior part of the intervertebral disc and of the neural arch [10,11]. Keller’s study conducted on elderly people showed that, at the level of the intervertebral discs affected by degenerative changes, there was a more even distribution of the load at the level of the vertebral plates compared to young population [17].

This behavior was also confirmed in the present study where the initial loading of the posterior side of the intervertebral disc and the back neural elements was noticed during the increasing loading process. The same effect was registered by Michio Hongo [18] within a biomechanical study where higher loads were generated at the level of the superior vertebral plate compared to the inferior plate. The structural changes at the level of the intervertebral disc and the vertebral plate favor load transfer in the posterior area, which can generate pain and spinal disc herniation [19].

When observing the changes generated at the level of the vertebral body and of the neural arch, it can be concluded that they were higher at the level of the superior vertebral plate (Figure 5a,b). According to the mechanostat theory [20,21], bone mass is influenced by the stresses to which it is subjected. Thus, in areas with an increased stress, bone growth is registered, while in less stressed areas, bone loss is registered. At least theoretically, the posterior vertebral area should be stronger compared to the anterior area. Even from the structural point of view, the posterior and the inferior areas of the vertebral body present more advantages. The vertebral trabeculae are more frequent in the posterior area of the vertebral body compared to the anterior area [22], and similarly, the trabecular system is thicker in its lower area [23]. These facts were also confirmed by Grant in his study [24]. All these aspects are also supported by the trabecular system orientation, which intersects in the posterior area of the vertebral body. Therefore, from the biomechanical point of view, the posterior area of the vertebral segment presents more advantages. This fact could also be observed in the present study. After reaching a peak loading value of T12–L1, Ll–L2, and of the posterior area of the vertebral body, the stress began to be distributed to the anterior area of the vertebral body. After reaching a maximum load on the intervertebral discs, the L1 trabecular system of the vertebral body was stressed and it gradually deformed. However, after reaching a maximum deformation and strength value of the superior and anterior area of the vertebral body, its trabecular system failed completely, generating the vertebral fracture (Figure 6a,b).

These findings are also confirmed by another biomechanical study conducted by Pollintine [25], where it was shown that degenerative changes of the intervertebral discs generate an increased loading of the anterior area of the vertebral body, which favors the prone fracture. These findings can explain why this area has an increased disposition towards fractures. Anterior wedge fracture is the most frequent pathology in the dorsal and lumbar areas in elderly populations [26] and is characterized by anterior compaction of the vertebral body without any harm to the posterior vertebral wall. Similar results were recorded using the finite-element method, where a minimum strength at the anterior area of the vertebral body could be observed [27]. Similar studies show that at the same time as intervertebral disc degeneration and reduced mineral density, a reduction of the strength of the anterior area of the vertebral plate can also be observed [28,29]. The reduced strength of the vertebral body makes it prone to gradual compaction and kyphosis occurrence [30]. Most vertebral body fractures are the result of a combination of forces which act both vertically and horizontally [31].

In this study, in both experimental situations a 2000 N load was gradually generated, but the fracture only occurred when the axial load of the vertebral body segment was generated. We can reason that this situation occurred because, for the axial and extension compression test, the posterior segments were mainly loaded, and thus a larger part of the force was taken over and absorbed by the deformation of the articular processes, and only after reaching the maximum capacity of the posterior vertebrae the load was taken over by the vertebral body. This biomechanical behavior is similar to the spine loading in extension, and thus it can be inferred that it can bear a bigger load in extension.

Osteoporotic spine fractures are produced in complex biomechanical and structural conditions which involve all the structures of the vertebral segment, starting with the vertebral body, neural arch, and ending with the ligamentous and capsular system, influenced by their degenerative changes.

The method chosen for this experimental study enabled the acquisition of information from many components of the vertebral segment in real time during compression loading. The advantage of this method was that the whole study was conducted experimentally, using optical methods, unlike studies using the finite-element method, where the difficulty of building the geometric model of the vertebral body (aspects of which can presently be eliminated by importing the model from the CT), is doubled by the important issue of the precision of the material data input. It is well known that both the vertebral body and the intervertebral discs are highly anisotropic, with different mechanical characteristics for different sections.

This method also presented disadvantages because the vertebrae had a complex structure without straight lines, which raised technical difficulties when it came to lighting the part to eliminate shadows. For this reason, the data obtained could have been altered due to dehydration of the intervertebral discs and the ligament system. Another limitation of this study was that there was only one specimen. The data obtained need to be confirmed by further studies, and larger cohorts are needed. This study method can also be used to analyze vertebral spine segment biomechanical behavior in the case of different techniques used in treatment for osteoporotic spine fractures (vertebroplasty, kyphoplasty).

## 5. Conclusions

This experimental method appears to be a valid technique for acquiring data regarding the biomechanical behavior of the osteoporotic spine and the mechanisms involved in osteoporotic spine fractures.

The behavior of the spine on axial compression forces was different if there was a pure axial load or an axial and extension load due to anatomical and structural vertebral bone and intervertebral discs characteristics. If there was an extension component of the compression force at the moment of the initial loading, part of this load was absorbed by the posterior column with higher mechanical resistance. After the maximum capacity of the absorption was reached, in both situations the behavior was similar, resulting in a vertebral body fracture. The mechanical properties of the aged intervertebral disc influenced the pattern of loading.
